# Planar Two-Dimensional Vibration Isolator Based on Compliant Mechanisms

**DOI:** 10.3390/mi16010010

**Published:** 2024-12-25

**Authors:** Ruizhe Zhu, Jinpeng Hu, Long Huang, Leiyu Zhang, Guangan Ren

**Affiliations:** 1 International College of Engineering, Changsha University of Science and Technology, Changsha 410114, China; shuruizhe1122@163.com; 2 School of Automotive and Mechanical Engineering, Changsha University of Science and Technology, Changsha 410114, China; 15798090198@163.com (J.H.); 369220424@163.com (G.R.); 3 Hunan Provincial Key Laboratory of Safety Design and Reliability Technology for Engineering Vehicle, Changsha University of Science and Technology, Changsha 410114, China; 4 College of Mechanical Engineering and Applied Electronics Technology, Beijing University of Technology, Beijing 100022, China

**Keywords:** compliant mechanisms, negative stiffness, positive stiffness, vibration isolator, dynamics

## Abstract

In practical engineering applications, the vibration is often generated in various directions and can be harmful to the engineering equipment. Thus, it is necessary to develop vibration isolators that can reduce vibration in multiple directions. In this paper, we propose a planar two-dimensional vibration isolator based on compliant mechanisms. The proposed mechanism consists of two negative stiffness-compliant modules and two positive stiffness-compliant modules, which leads to the quasi-zero stiffness (QZS) property in the mechanism. The dynamic model is established by using the third-order Taylor expansion and the harmonic balance method. Based on the dynamic model, the influence of different parameters on the displacement transmissibility is discussed, including damping ratio, system stiffness, and excitation amplitude. Finally, we conducted the vibration isolation experiments and obtained the displacement transmissibility of the isolator. The results verify that the proposed isolator has good isolation performance for low-frequency vibration.

## 1. Introduction

A passive vibration isolator with quasi-zero stiffness (QZS) is a topic of wide concern in the field of vibration isolating [1,2], which has the advantages of a simple structure, low cost, and low requirements for maintenance. The QZS vibration isolator possesses the nonlinear character of low dynamic stiffness and high static stiffness, which can greatly reduce the natural frequency of the system while maintaining a high load-bearing capacity, thus extending the vibration isolation frequency band into the low-frequency region. To date, researchers have developed various types of QZS vibration isolators, including oblique-springs-based QZS vibration isolators, cam-mechanism-based QZS vibration isolators, and compliant-mechanism-based QZS vibration isolators.

For oblique-springs-based QZS vibration isolators, Carrella et al. designed many types of passive QZS vibration isolators based on oblique strings [3,4,5]. Wei developed a 1DOF QZS isolator with two transversal springs and one portrait spring [6]. Xu et al. designed the TSC model isolator with five springs and analyzed the amplitude–frequency characters using the harmonic balance method [7]. Zhou et al. designed a QZS vibration isolator with cam-roller-spring mechanisms and analyzed the dynamic performance based on a piecewise nonlinear dynamic model [8]. Zhang et al. designed a vibration isolation system based on a pair of oblique springs or a spring-rod mechanism as a negative stiffness mechanism [9]. Thakadu et al. proposed a passive vibration isolator that integrated a dynamic vibration absorber with a negative stiffness spring and investigated the dynamic behavior by force and displacement transmissibility equations [10]. Zhou et al. designed a novel combination of a V-shaped lever, plate spring, and cross-shaped structure vibration isolation platform and obtained displacement transmissibility by the average method [11].

For cam-mechanism-based QZS vibration isolators, Li et al. designed many types of passive QZS vibration isolators based on cam mechanisms [12,13,14]. Tan et al. designed a device capable of customizing nonlinear forces based on the analysis of polynomial nonlinear forces and piecewise nonlinear forces and verified the result by measuring the actual nonlinear forces [15]. Li et al. developed a cam-roller-springs isolator that has the character of low dynamic stiffness and high static stiffness and completed the dynamic analysis based on the averaging method [16]. Sun et al. described a vibration isolator with a novel parabolic-cam-roller negative stiffness mechanism which expresses the character of low dynamic stiffness and high static stiffness, completed dynamic analysis based on Lagrange equations, and verified its amplitude–frequency characters using the harmonic balance method [17]. Zhou et al. proposed many types of passive QZS vibration isolators based on cam mechanisms [18,19,20].

For compliant-mechanism-based QZS vibration isolators, Sui et al. developed a QZS isolator based on an inclined trapezoidal beam and investigated the nonlinear stiffness through finite element simulation [21]. Ding et al. designed a rigid–flexible coupling QZS vibration isolator with high-static–low-dynamic stiffness characteristics [22]. Zhang et al. propose a compliant QZS isolator to isolate low-frequency torsional vibrations in the shaft system. Fan et al. proposed a metastructure consisting of numerous unit cells with QZS character [23]. Yu et al. designed a new QZS-compliant shock isolator based on a fixed-guided beam with negative stiffness and a Roberts configuration compliant beam with positive stiffness, analyzed and calculated theoretically using an elliptic integration method, and verified by comparison using finite element simulation [24]. Lu et al. designed a compliant curved beam support for a near-zero frequency vibration isolator and revealed the stiffness modulation mechanism by the static analysis [25]. Fan et al. developed a metastructure consisting of numerous unit cells with quasi-zero stiffness properties and studied the vibration isolation performance of the metastructure by theoretical and simulation methods [26].

The prior QZS isolators have excellent vibration isolation performance in the vertical direction; however, in practice, the vibration is often generated in various directions; thus, it is necessary to develop vibration isolators that can reduce vibration in multiple directions. Aiming at this goal, Jing et al. proposed many types of multiple DOF vibration isolators based on the X-shaped linkage mechanism [27,28,29]. Zhu et al. developed a three-degree-of-freedom QZS vibration isolator model and its inverse construction method based upon the target restoring force functions [30]. Wang et al. proposed a multi-direction QZS vibration isolator based on the designed geometrical relationship between the torsion spring and the linear spring [31]. Zhou et al. also developed many types of multiple DOF vibration isolators [18,32,33]. Liu et al. developed a nonlinear passive vibration isolator composed of a linear spring in parallel with the Euler buckled beams and obtained the frequency response curves by using the Harmonic Balance Method for both force and displacement excitations [34].

In this paper, we propose a planar two-dimensional vibration isolator based on compliant mechanisms. It has its unique advantage: the compliant-mechanism-based property enables the isolator to be manufactured through 3D printing, and it is easy to miniaturize. The rest of the paper is organized as follows. Section 2 introduces the design principle of a planar two-dimensional quasi-zero-stiffness vibration isolator. Section 3 describes the static experiments. In Section 4, the dynamic analysis of the QZS vibration isolator is conducted. In Section 5, the vibration isolation experiments are conducted.

## 2. Design of Planar Two-Dimensional Quasi-Zero-Stiffness Vibration Isolator

Aiming at the vibration isolation in two directions on the horizontal plane, we propose a novel two-dimensional QZS vibration isolator based on the combination of positive and negative stiffness-compliant mechanisms. The positive stiffness-compliant mechanism is constructed based on the double parallelogram compliant mechanism, and the negative stiffness-compliant mechanism is constructed based on a cosine-curve beam.

### 2.1. Negative Stiffness-Compliant Module Based on Cosine-Curve Beams

**Figure 1:**
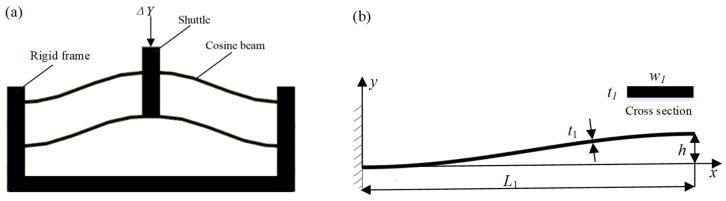
(**a**) Negative stiffness compliant mechanism based on the cosine-curve beams; (**b**) the cosine-curve beam.

The bistable mechanism is usually used to provide negative stiffness. When the bistable mechanism is under external loads, it first exhibits positive stiffness, then exhibits negative stiffness, and finally exhibits positive stiffness. Herein, we use the combination of the cosine-curve compliant beams to construct the bistable mechanism, as shown in Figure 1a. The cosine-curve beam contains 0.5 cycles, which is expressed by the following equation

(1) $$y(x)=\frac{h}{2}\left[1-\cos(\frac{\pi x}{L_{1}})\right]\;\;\begin{array}{c} x\in \left[0,L_{1}\right] \end{array},$$

where $L_{1}$ denotes the length of the negative stiffness cosine-curve beam along the *X*-axis, and *h* denotes the amplitude of the negative stiffness cosine-curve beam.

To ensure that the end of the cosine-curve beam generates displacement only in the Y-direction, the proposed negative stiffness compliant mechanism adopts a symmetric arrangement, where the two layers of cosine-curve beams are connected by a rigid frame; the high stiffness of the frame provides a fixed support boundary condition for the cosine-curve beams, thus avoiding horizontal stretching of the cosine-curve beams during the deformation process. According to Ref. [35], if $h/t_{1}\geq 6$, when the external force in the Y-direction acts on the single cosine-curve beam, it exhibits negative stiffness in the displacement range $\left[8{t_{1}}^{2}/3h,2h-8{t_{1}}^{2}/3h\right]$, and the absolute value of the negative stiffness can be expressed as

(2) $$K_{1}=4\pi^{4}Ew_{1}{t_{1}}^{3}h/[{{48L}_{1}}^{3}\left(0.67h-8{t_{1}}^{2}/3h\right)],$$

Thus, the absolute value of the total negative stiffness for the compliant mechanism shown in Figure 1 can be expressed as

(3) $$K_{-}={4K}_{1}$$

### 2.2. Positive Stiffness-Compliant Module Based on Parallelogram Mechanisms

**Figure 2:**
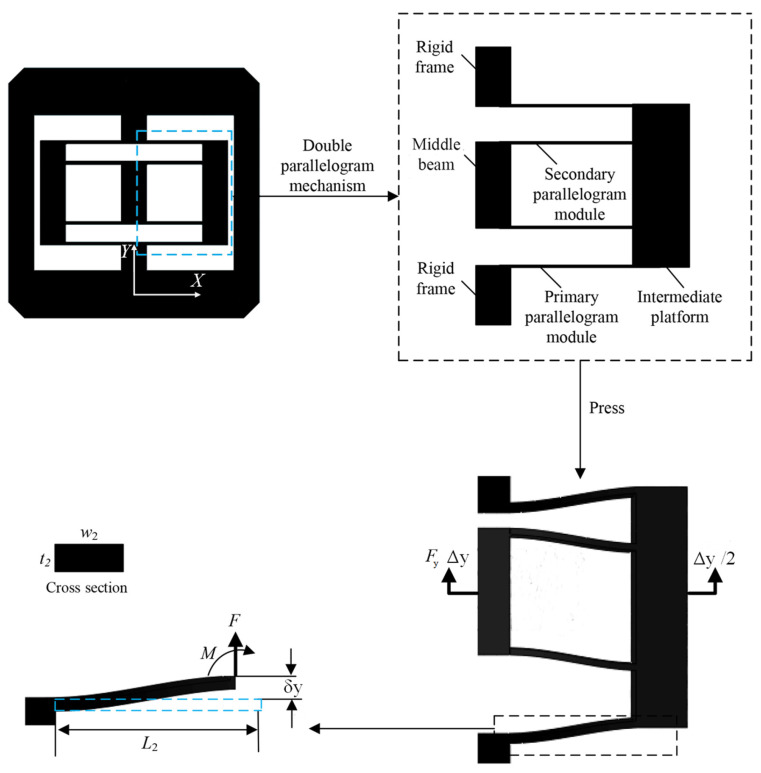
Double parallelogram compliant mechanism.

Parallelogram-based compliant mechanisms are usually used to generate translation, which possesses positive stiffness. To realize the translation in a single direction, here we use a symmetric parallelogram mechanism as the positive stiffness module, which is constructed by connecting four parallelogram mechanisms in series and parallel, as shown in Figure 2. If the rigid frame is fixed, and an external force is applied perpendicular to the middle beam, the motion pattern of the middle beam is the translation along the Y-direction.

In this circumstance, according to their rotation angles and the boundary conditions [36], the stiffness of the double parallelogram mechanism (Figure 2) in Y-direction can be obtained as

(4) $$K_{2}=\frac{F_{y}}{\Delta y}=\frac{2F}{2\delta y}=\frac{Ew_{2}{t_{2}}^{3}}{{L_{2}}^{3}},$$

Therefore, the stiffness of the whole module can be obtained as

(5) $$K_{+}=2K_{2},$$

**Figure 3:**
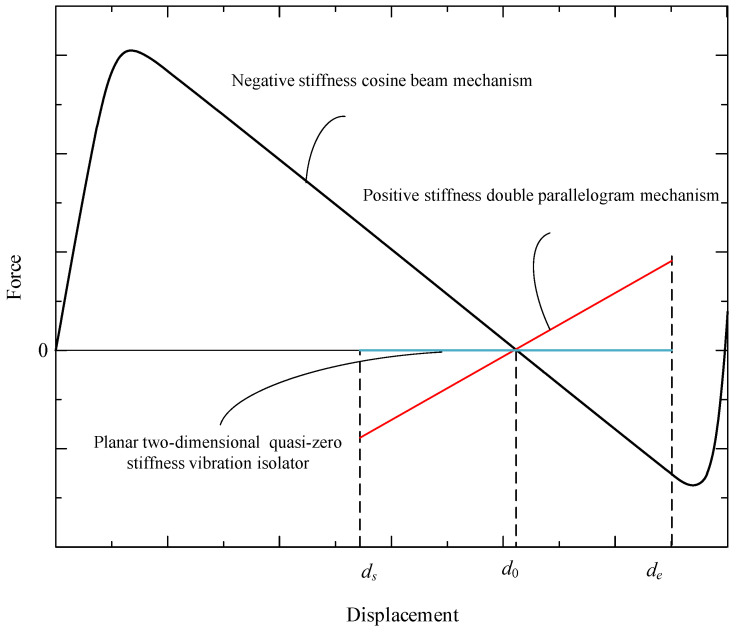
Force–displacement curves of planar two-dimensional quasi-zero-stiffness vibration isolator.

Here, the negative-stiffness cosine-beam mechanism is pre-compressed to the configuration corresponding to zero restoring force. Meanwhile, the pre-compressed cosine beam has negative stiffness. Thus, the mechanism combined with the negative stiffness characteristic and the positive stiffness characteristic can achieve QZS within a certain displacement range ($d_{s}$,${\;d}_{e}$), as shown in Figure 3.

### 2.3. Overall Design of Planar-Dimensional Quasi-Stiffness Vibration Isolator

**Figure 4:**
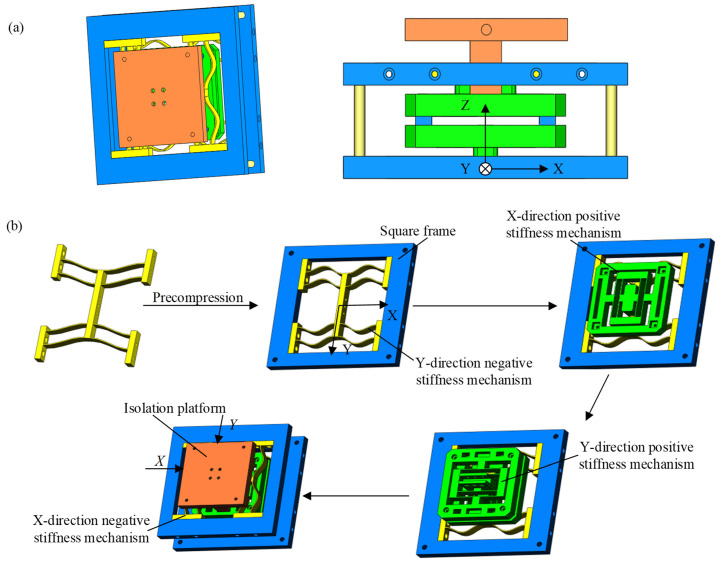
Model of planar two-dimensional quasi-zero-stiffness vibration isolator. (**a**) General diagram of planar two-dimensional quasi-zero-stiffness vibration isolator; (**b**) component diagram of planar two-dimensional quasi-zero-stiffness vibration isolator.

Based on the negative and positive stiffness module, here we propose the innovative design of the planar two-dimensional QZS vibration isolator, as shown in Figure 4. The vibration isolator contains four layers of compliant mechanisms, which contribute to the compact structure. The bottom and top layers are negative stiffness-compliant mechanisms in the Y-direction and X-direction, while the intermediate layers are positive stiffness-compliant mechanisms in the Y-direction and X-direction.

The negative-stiffness compliant mechanism consists of two symmetrically connected negative-stiffness modules. They are pre-compressed by displacement $d_{0}$ so that they both exhibit negative stiffness, then they are embedded in the rigid square frame and fixed together. In this way, the absolute value of the stiffness is $2K_{-}$. The square frames in the bottom and top layers are fixed together by four rigid columns.

The intermediate layers use the parallelogram-based module directly. The middle beam of the parallelogram-based module is fixed with the shuttle of the negative stiffness module. The rigid frame of the positive stiffness modules is fixed together with four rigid columns that are connected to a platform, which serves as the output of the whole system. The platform has two decoupled translational degrees of freedom, namely the X-direction and Y-direction.

In this paper, the required constant force for the vibration isolator is 0 N, and the required region for the constant force is [−5 mm, 5 mm]. Considering the dimension of the structure and manufacture precision, $L_{1}$ and $L_{2}$ for the negative and positive stiffness modules are determined to be 40 mm and 13.5 mm, while w and t for both modules are determined to be 10 mm and 1 mm. To enable large deformation, polypropylene is adopted as the 3D printed material of the compliant beams, whose Young’s modulus is 220 MPa. The detailed parameters of positive and negative beams are listed in Table 1. According to the equations listed in Section 2.1 and Section 2.2, the stiffness of the vibration isolator is a small positive stiffness, namely 0.0522 N/mm, and the restoring force of the platform is within the range [−0.261 N, 0.261 N].

## 3. Static Experiments of Planar Two-Dimensional Vibration Isolator

The compliant components in positive and negative stiffness modules were 3D-printed with polypropylene material, while the rigid components were 3D-printed with polylactic acid material. Considering 3D-printed materials’ hysteresis, we conducted loading and unloading experiments repeatedly and found the hysteresis was not obvious and it could basically recover to its initial state. The parameters of the materials are shown in Table 2.

**Figure 5:**
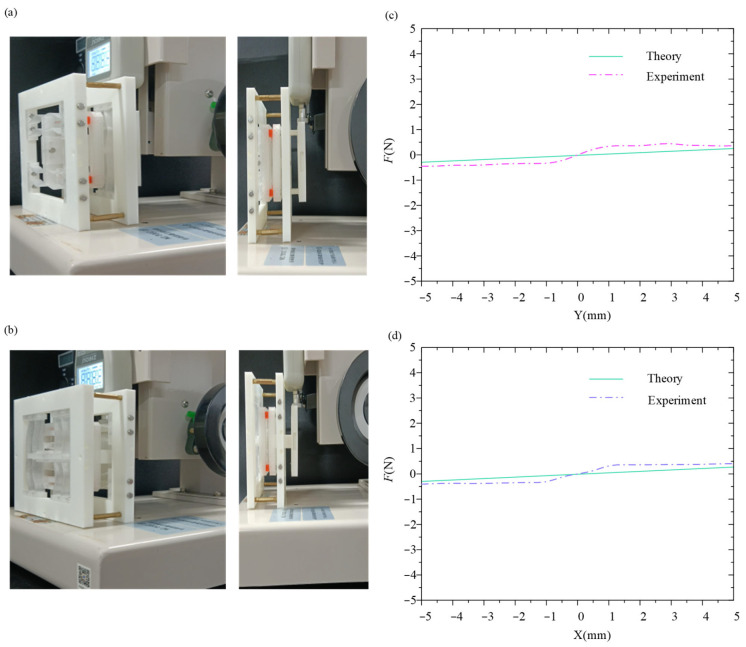
(**a**,**b**) Experimental setup for X-direction and Y-direction. (**c**,**d**) Comparison of theoretical and experimental force-displacement curves in X-direction and Y-direction.

After we assembled the vibration isolator prototype, we established the static experimental setup for the vibration isolator, as shown in Figure 5a,b, and conducted static compression experiments in ±X-direction and ±Y-direction. The comparison of theoretical and experimental force-displacement curves in both directions is shown in Figure 5c,d. It can be observed that the experimental curves are in agreement with the theoretical curves in a large displacement range. The largest error between theoretical and experimental results is within 0.3 N.

## 4. Dynamics of the Quasi-Zero-Stiffness Vibration Isolator

In this section, the experimental force–displacement curves are expressed with the third-order Taylor expansion, which can more accurately fit the region near the equilibrium point. We also solved the steady-state response of the system using the harmonic balance method.

Due to the symmetrical arrangement of modules, the QZS characteristics of the mechanism in the X-axis and Y-axis are approximately the same. Here, we conduct the dynamic analysis in the Y-axis, for instance. The restoring force of the experimental force-displacement curve is expanded to the third-order Taylor expansion at $∆Y_{0}=0$, and the simplified force-displacement expression is obtained as

(6) $$F_{app}\left(\Delta Y_{0}\right)=\sum_{n=0}^{3}\frac{1}{n!}F^{\left(n\right)}{\left(\Delta Y_{0}\right)}^{n}=a_{3}\Delta {Y_{0}}^{3}+a_{2}\Delta {Y_{0}}^{2}+a_{1}\Delta Y_{0}+a_{0},$$

where $Y_{0}$ is excitation amplitude, $a_{3}=4.354\times 10^{-3}$, $a_{2}=-1.195\times 10^{-4}$, $a_{1}=0.01771$, and $a_{0}=-2.008\times 10^{-3}$.

Differentiating Equation (6) yields the approximate stiffness expression obtained as

(7) $$k_{app}(\Delta Y_{0})=3a_{3}\Delta {Y_{0}}^{2}+2a_{2}\Delta Y_{0}+a_{1},$$

**Figure 6:**
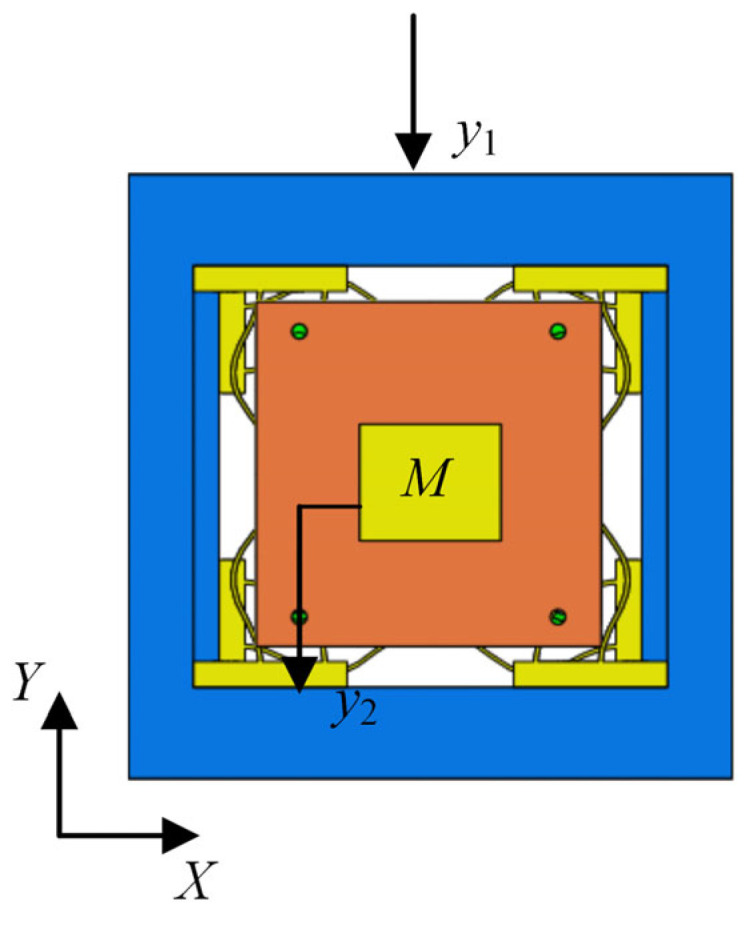
Displacement transmission structure diagram of vibration isolator in *Y*’s direction.

Assuming that the translational displacement excitation in the *Y*-axis is applied on the square frame, the QZS vibration isolation can be reduced to a second-order single-degree-of-freedom nonlinear system with a mass loading of M. The vibration isolation system is in the static equilibrium position initially, as shown in Figure 6.

If the simple harmonic displacement excitation applied on the square frame is expressed by $y_{1}=Y_{0}\sin wt$, we can express the system’s dynamic equation as

(8) $$M{\ddot{y}}_{2}+c\left({\dot{y}}_{2}-{\dot{y}}_{1}\right)+F_{app}\left(y_{2}-y_{1}\right)=0,$$

Substituting Equation (6) into Equation (8) yields

(9) $$M\ddot{y}+c\dot{y}+a_{3}y^{3}+a_{2}y^{2}+a_{1}y+a_{0}=MY\omega^{2}\sin\left(\omega \;t+\varphi \right),$$

where y is the relative displacement between the isolation platform and the square frame under excitation and

(10) $$\left\{\begin{array}{c} y=y_{2}-y_{1}\\ \dot{y}={\dot{y}}_{2}-{\dot{y}}_{1}\\ \ddot{y}={\ddot{y}}_{2}-{\ddot{y}}_{1} \end{array}\right.,$$

Assuming that $\Omega =\omega /\omega_{n}$, $t=\tau /\omega_{n}$, $\hat{r}\left(\tau \right)=y$, ${\hat{r}}^{\prime }\left(\tau \right)=\dot{y}/\omega_{n}$, ${\hat{r}}^{\prime \prime }\left(\tau \right)=\dot{y}/{\omega_{n}}^{2}$, ξ= c/2 M$\omega_{n}$, $\chi_{3}=a_{3}/a_{1}$, $\chi_{2}=a_{2}/a_{1}$, $\chi_{1}=1$, $\chi_{0}=a_{0}/a_{1}$ and $\omega_{n}=\sqrt{a_{1}/M}$; Ω denotes the non-dimensional vibration excitation frequency, and $\omega_{n}$ denotes the intrinsic frequency; therefore Equation (10) can be reduced to

(11) $${\hat{r}}^{\prime \prime }\left(\tau \right)+2\xi {\hat{r}}^{\prime }\left(\tau \right)+\chi_{3}{\hat{r}}^{3}\left(\tau \right)+\chi_{2}{\hat{r}}^{2}\left(\tau \right)+\chi_{1}\hat{r}\left(\tau \right)+\chi_{0}=Y_{0}\Omega^{2}\sin\left(\Omega \tau +\varphi \right),$$

The simplified differential equation is the typical Duffing equation to describe the nonlinear vibration behavior. Here, we use the harmonic balance method to obtain its approximate solution. The steady-state response solution of the system can be set as $\hat{r}(\tau )=A\sin\left(\Omega \tau \right)$, where A denotes the amplitude.

Substituting the established steady-state response solution into Equation (11) yields

(12) $$\left(0.75\chi_{3}A^{3}-\Omega^{2}A+A\right)\sin\left(\Omega \tau \right)-2\xi \Omega A\cos\left(\Omega \tau \right)\;-0.25\chi_{3}A^{3}\sin\left(3\Omega \tau \right)+0.5\chi_{2}A^{2}\left[1-\cos\left(2\Omega \tau \right)\right]+\chi_{0}\;=Y_{0}\Omega^{2}\cos\phi \sin\left(\Omega \tau \right)+Y_{0}\Omega^{2}\sin\phi \cos\left(\Omega \tau \right),$$

Ignore the higher-order terms in Equation (12), and extract the coefficients of sin⁡(Ωτ) and cos⁡(Ωτ) in both sides of the equation, then we obtained

(13) $$\left\{\begin{array}{c} 0.75\chi_{3}A^{3}-\Omega^{2}A+A=Y_{0}\Omega^{2}\cos\phi \\ -2\xi \Omega A=Y_{0}\Omega^{2}\sin\phi \end{array}\right.,$$

Then, we can obtain the amplitude–frequency characteristic equation and phase-frequency characteristic equation

(14) $$\left\{\begin{array}{c} {\left(\frac{3}{4}\chi_{3}A^{3}-\Omega^{2}A+A\right)}^{2}+{\left(-2\xi \Omega A\right)}^{2}={\left(Y_{0}\Omega^{2}\right)}^{2}\\ \cos\varphi =(\frac{3}{4}\chi_{3}A^{3}-\Omega^{2}A+A)/(Y_{0}\Omega^{2}) \end{array}\right.,$$

**Figure 7:**
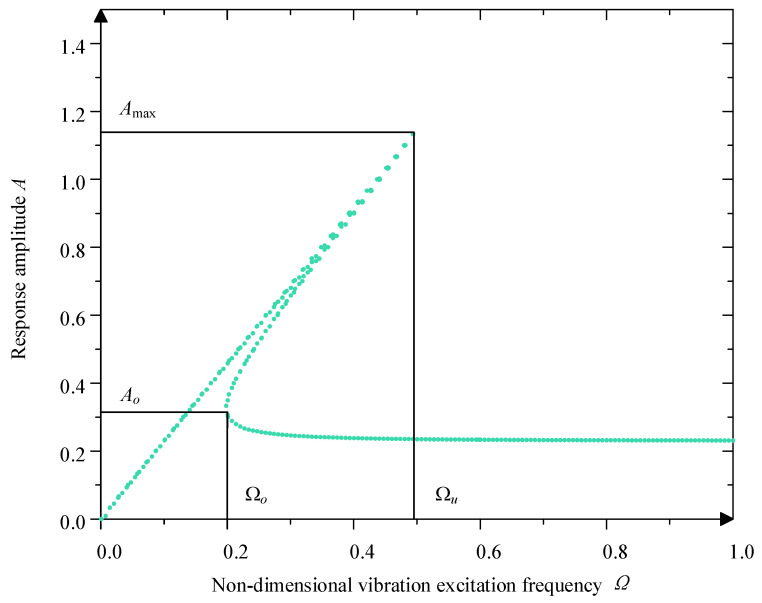
Amplitude–frequency character curve.

It can be inferred from Equation (14) that the system’s response amplitude is mainly about the damping ratio ξ, system stiffness $\chi_{3}$ and excitation amplitude $Y_{0}$, which together determine the system’s dynamic response characteristics at different frequencies. According to the structural parameters of the designed vibration isolation mechanism, ξ=0.05. $\chi_{3}=0.250$. $Y_{0}=0.230$ are selected, and its amplitude–frequency characteristic curve is plotted, as shown in Figure 7.

From Figure 7, the mechanism exhibits obvious jumping behavior, which is one of the characteristics of nonlinear systems. Within a certain excitation frequency interval, the response at the same frequency corresponds to multiple amplitude solutions. In the Figure 7, $\Omega_{0}$ is the upward jump frequency, and $\Omega_{u}$ is the downward jump frequency. In this interval, when the value of Ω gradually increases, the value of A changes along the upper branch of the curve; when the frequency increases to $\Omega_{u}$, the value of A reaches the peak value $A_{max}$ and suddenly jumps to the corresponding point of the lower branch of the curve. As the value of Ω continues to increase to infinity, the value of *A* will gradually decrease. On the contrary, when sweeping from high frequency to low frequency, the value of A changes along the lower branch of the curve; when decreasing to $\Omega_{0}$, the value of A will jump to the value of the corresponding point of the lower branch of the curve. When decreasing to the value of $\Omega_{0}$, the value of A will jump to the value of the corresponding point of the upper branch of the curve again. At this time, the corresponding response amplitude is $A_{0}$.

Based on the above analysis, it can be seen that the jump phenomenon and the response amplitude are relative to the $\chi_{3}$, Y and ξ. So, analysis of the performance is based on these parameters.

**Figure 8:**
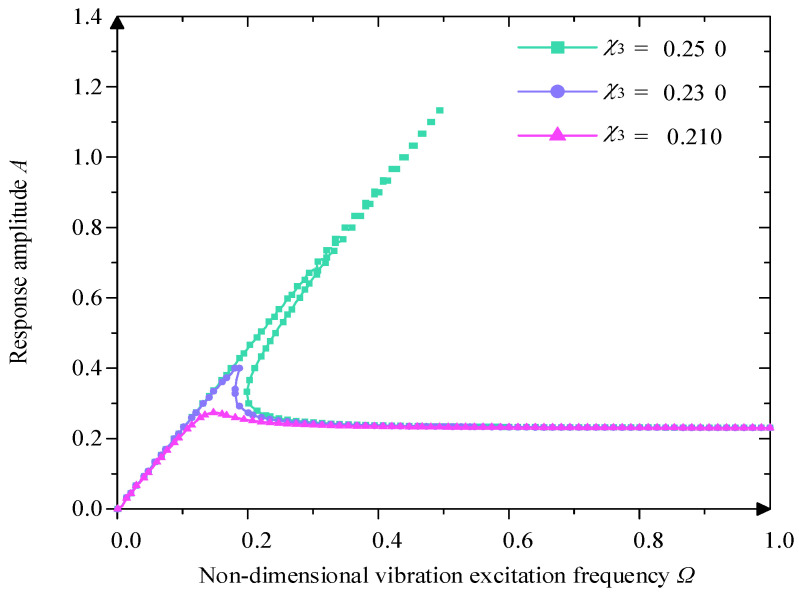
Influence of different stiffness $\chi_{3}$ on amplitude–frequency character curve.

From Figure 8, when $Y_{0}=0.230$ and the ξ=0.05, with the increase of stiffness, the value of A gradually increases, and its amplitude–frequency characteristic curve bends to the right; At the same time, the value of $\Omega_{0}$ and $\Omega_{u}$ increases significantly, and the jumping frequency interval also increases gradually, and the vibration isolation band of the system becomes narrower, which is not conducive to the low-frequency vibration isolation. Therefore, the stiffness is not suitable to be set too large, and in the high-frequency region, the stiffness has little effect on its amplitude–frequency characteristic curve.

**Figure 9:**
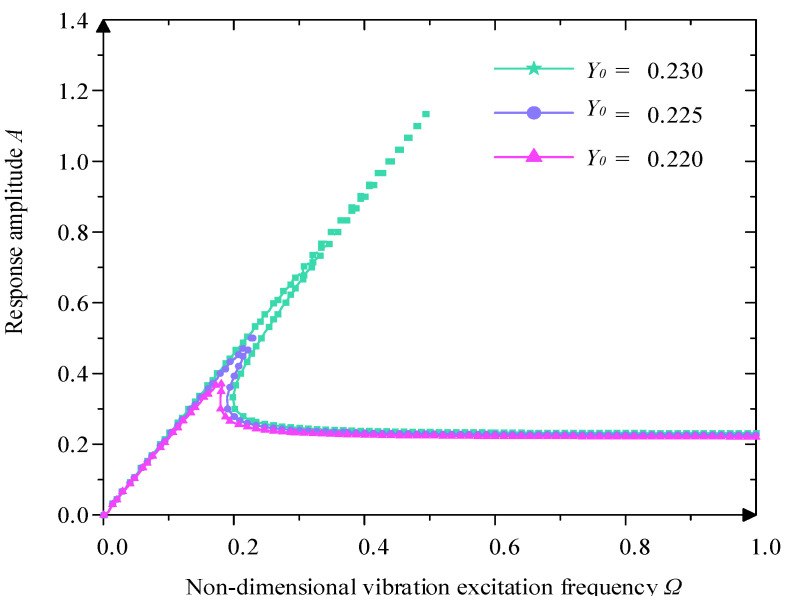
Influence of different excitation amplitude $Y_{0}\;$on amplitude–frequency character curve.

When $\chi_{3}=0.250$ and ξ=0.05, from Figure 9, when the value of Ω increases gradually, the stabilized response amplitude of the system also grows, which indicates that the strength of the excitation has a direct influence on the dynamic response of the system. Meanwhile, the shape of the amplitude–frequency characteristic curve also changes, the bending degree becomes more obvious, its upward and downward jumping frequency region also increases gradually, and its increasing downward jumping frequency reflects the aggravation of the system’s nonlinear characteristics.

**Figure 10:**
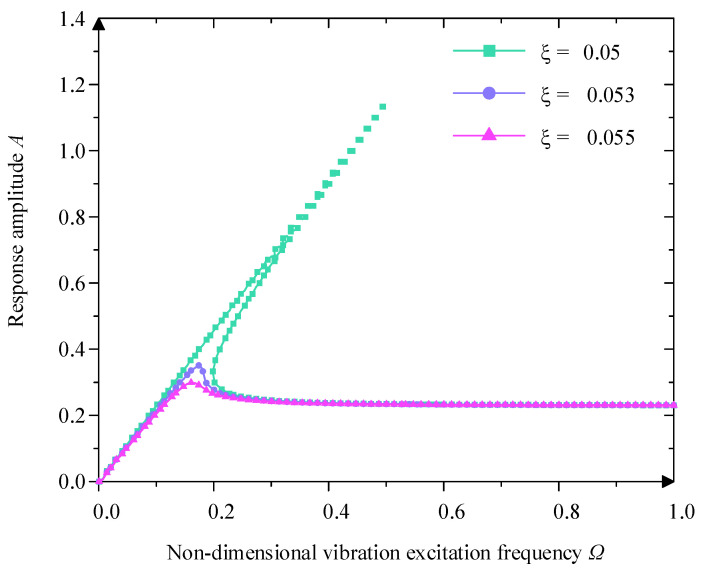
Influence of different damping ratio  ξ on amplitude–frequency character curve.

When the $Y_{0}=0.230$ and $\chi_{3}=0.250$, from Figure 10, ξ mainly influences the upper and lower jump frequencies. With the increase of ξ, the value of A gradually decreases, the value of $\Omega_{0}$ and $\Omega_{u}$ are reduced, the jump frequency interval is also gradually narrowed down, and at the same time, the jumping phenomenon and the unstable region gradually disappear because of the increase of the damping ratio, and at high excitation frequency, the influence of the damping ratio is small. Therefore, a larger damping ratio can eliminate the jump phenomenon and obtain a better vibration isolation effect.

Displacement transmissibility is the ratio of the displacement response amplitude to the excitation displacement amplitude through the vibration control system during the cycle of excitation and response. The displacement transmissibility is commonly used in the vibration isolation mechanism to evaluate the vibration isolation performance under vibration control. The displacement transmissibility formula of the planar two-dimensional QZS vibration isolation mechanism is as follows

(15) $$T_{a}=\frac{\left|Y_{0}+y\right|}{y}=\frac{\sqrt{A^{2}+Y_{0}^{2}+2AY_{0}\cos\varphi }}{Y_{0}},$$

According to Equation (15), the displacement transmissibility of the planar two-dimensional QZS isolation mechanism is mainly related to the ξ, $\chi_{3}$ and $Y_{0}$. A low displacement transmissibility means that the vibration isolation system can effectively isolate vibration and protect sensitive equipment from vibration.

**Figure 11:**
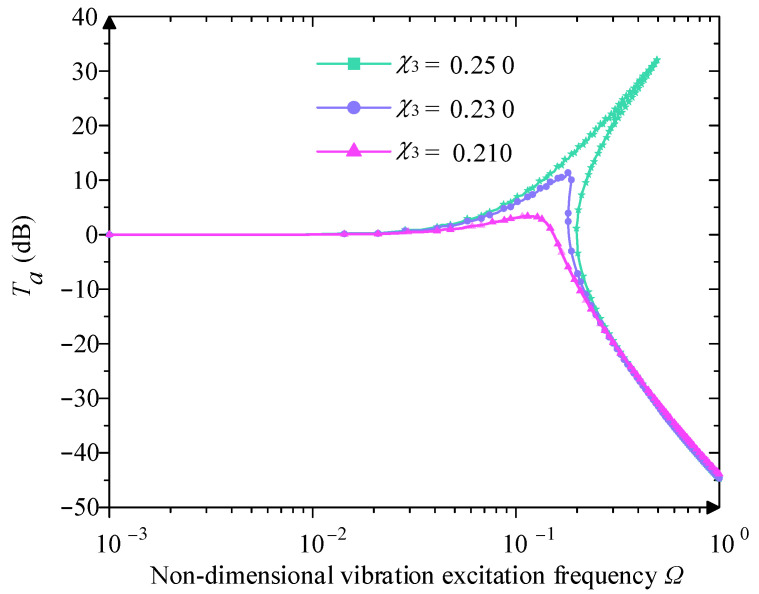
Influence of different system stiffness $\chi_{3}$ on displacement transmissibility.

When $Y_{0}=0.230$ and ξ=0.05, the paper analyses the relationship between $\chi_{3}$ and $T_{a}$, as shown in Figure 11. With the increase in the value of $\chi_{3}$, the displacement transmissibility curve of the vibration isolation mechanism bends to the right, the peak value of $T_{a}$ increases significantly, the jump phenomenon is more obvious, and the value of $\Omega_{0}$ and $\Omega_{u}$ increases gradually, and the frequency domain of vibration isolation decreases so that the low-frequency vibration isolation performance becomes worse, but the stiffness of the high-frequency region has less influence on the vibration isolation system. Therefore, in the design of the QZS vibration isolation mechanism, selecting a smaller value of stiffness is conducive to improving the low-frequency vibration isolation effect.

**Figure 12:**
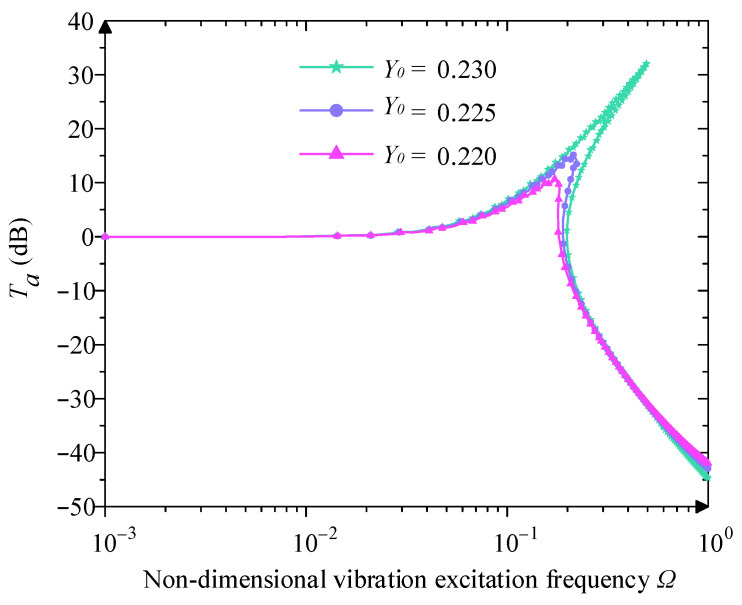
Influence of difference excitation amplitude $\;Y_{0}$ on displacement transmissibility.

When the $\chi_{3}=0.250$ and ξ=0.05, it analyses the relationship between $Y_{0}$ and $T_{a}$ according to Figure 12. As the value of Ω increases, the peak value of $T_{a}$ increases accordingly. This means that the displacement response at the output of the system is more significant at higher excitation amplitudes, which may lead to a worse performance of vibration isolation. Meanwhile, the initial isolation frequency also increases, which indicates that the frequency threshold of initial vibration isolation becomes higher, and the low-frequency isolation capability is weakened. In some cases, the displacement transmissibility curve may exhibit a jump phenomenon. The occurrence of jumps may lead to instability of the vibration isolation system, especially in the low-frequency domain.

**Figure 13:**
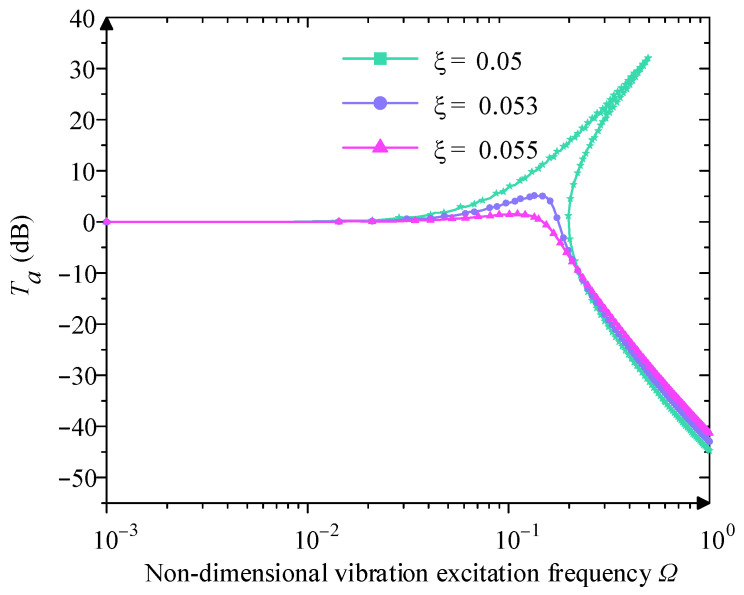
Influence of difference damping ratio ξ on displacement transmissibility.

When the $Y_{0}=0.230$ and $\chi_{3}=0.250$, analyze the relationship between ξ and $T_{a}$. From Figure 13, with the increase of the value of ξ, the peak value of $T_{a}$ decreases gradually, the initial isolation frequency decreases, the isolation frequency domain widens gradually, and the jump region also decreases gradually. In the low-frequency region, increasing the damping ratio is conducive to the improvement of the vibration isolation effect, but in the high-frequency region, the increase in the damping ratio has little effect on its vibration isolation performance.

## 5. Vibration Experiments

### 5.1. Experimental Setup

**Figure 14:**
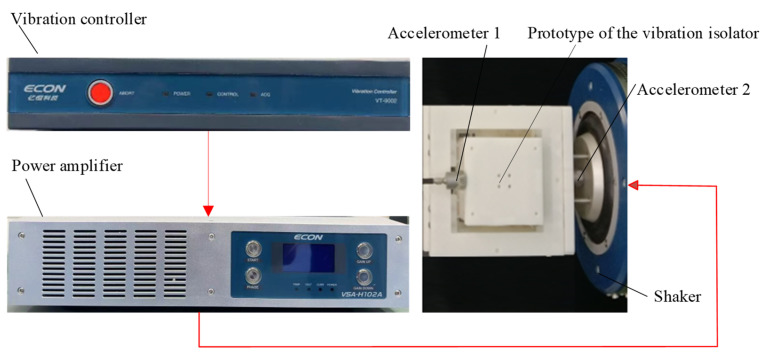
Vibration isolation experimental platform.

The vibration experimental setup is shown in Figure 14. The vibration isolation performance of the vibration isolator is verified under different excitations. The vibration experiments in the X and Y directions are performed separately. The Shaker is closed-loop controlled, and the control signal was output from the controller (ECON VT-9002), which was then amplified by the power amplifier (ECON VSA-H102A) to drive the shaker (ECON VE-5150). The rigid frame of the vibration isolator is fixed to the moving platform of the shaker. Two accelerometers (sensitivity of 100 mV/g) are fixed to the platform of the shaker and the output platform of the isolator. One is used to measure the excitation acceleration to form a closed-loop control, and the other is used to measure the acceleration response of the vibration isolator. The accelerometer signals were transmitted through the controller to a computer for further analysis.

### 5.2. Experimental Results

**Figure 15:**
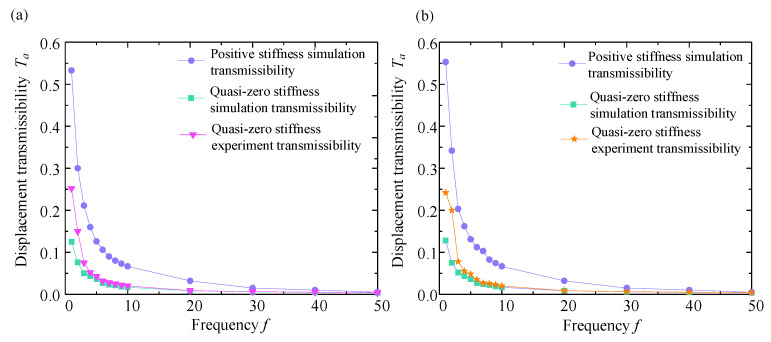
Displacement transmissibility curve when excitation amplitude is 3 mm. (**a**) Transmissibility curve diagram in direction X; (**b**) transmissibility curve diagram in direction Y.

**Figure 16:**
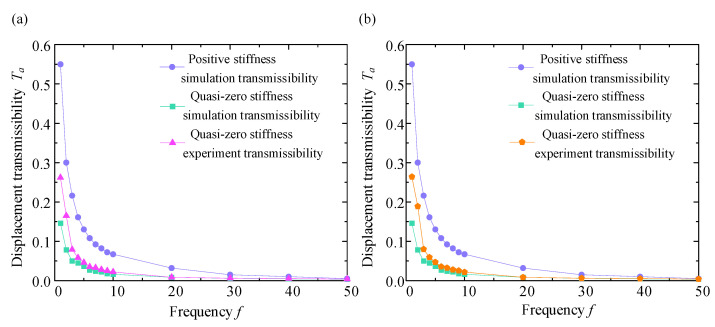
Displacement transmissibility curve when excitation amplitude is 5 mm. (**a**) Transmissibility curve diagram in direction X; (**b**) transmissibility curve diagram in direction Y.

To test the vibration isolation performance of the QZS vibration isolation system under sinusoidal displacement excitation, two different displacement excitations (3 mm and 5 mm) were selected for the experiment, and a series of vibration tests were carried out at these amplitudes, respectively. Under the premise of conforming to the parameter settings of the excitation platform, the frequency setting of the platform was extended from 1 Hz to 50 Hz and adjusted once every 1 Hz interval. The sinusoidal excitation test allows continuous recording of the response of the vibration isolation system at a series of frequencies, starting with 1 Hz vibration, and recording the output signal data of the acceleration sensor 2 after the system response has stabilized. The displacement transmissibility curves obtained at different displacement excitation amplitudes are displayed in Figure 15 and Figure 16.

From Figure 15 and Figure 16, within the frequency range of the excitation, we can obtain the exact value of $T_{a}$ at a specific frequency. When the excitation amplitude is 3 mm, the maximum simulated value of the $T_{a}$ is 0.13, the maximum experimental value of $T_{a}$ is 0.25, and the maximum value of $T_{a}$ of the linear positive stiffness system is 0.53. Moreover, the value of $T_{a}$ decreases with the increasing value of f. When the excitation amplitude is 5 mm, the maximum simulated value of $T_{a}$ is 0.15, and the maximum experimental value of $T_{a}$ is 0.262, while the maximum value of $T_{a}$ of the linear positive stiffness system is 0.55, and the value of $T_{a}$ also decreases with the increasing value of f. In the excitation frequency range of 1 Hz to 50 Hz, the transmissibility curve of the linear positive stiffness system is nearly above the QZS transmissibility curve, and this is more obvious in the low-frequency range of 1 Hz to 10 Hz. When the excitation frequency gradually increases, the displacement transmissibility curves of the two gradually approach, and they are nearly the same above 30 Hz.

As the excitation amplitude increases, the maximum value of $T_{a}$ also tends to increase, and the value of $T_{a}$ also increases accordingly. However, as the value of f gradually increases, the transmissibility curve of the vibration isolation mechanism decreases regardless of the excitation amplitude, and this trend of transmissibility change is consistent with theoretical analysis. In the theoretical analysis of displacement transmissibility, the transmissibility curve first increases and then decreases with a resonance peak. In the actual experiment, the starting excitation frequency starts at 1 Hz to avoid system resonance, so only the curve on the right side of the peak is obtained.

Meanwhile, the result of vibration isolation experiments shows a good match with the numerical simulation transmissibility curves, which not only verifies the correctness of the theoretical calculation and analysis but also the reliability of the experimental data. For both simulation and experimental data, the transmissibility of the QZS mechanism is much lower than that of the linear positive stiffness, and the vibration isolation effect of the QZS mechanism is better, meeting the design requirements.

## 6. Conclusions

In this paper, a planar two-dimensional vibration isolator is constructed by combining two parallelogram-based positive-stiffness compliant mechanisms and two curved-beam-based negative-stiffness compliant mechanisms. In comparison with the prior multi-DOF QZS vibration isolator, the proposed structure has the following advantages: the compliant-mechanism-based property enables the isolator to be manufactured through 3D printing, and it is easy to miniaturize. Then, we use the third-order Taylor expansion to obtain the experimental force–displacement curve and use the harmonic balance method to obtain the solution of the steady-state response. Based on the dynamic model, it is easy to analyze the influence of parameters $Y_{0}$, $\chi_{3}$, ξ on the amplitude-frequency character and obtain the answer that it is necessary to reduce $\chi_{3}$ and $Y_{0}$ and increase ξ reasonably to weaken the jumping phenomenon and isolate vibration in low-frequency environments. Furthermore, we conducted vibration isolation experiments on the shaker. The result of the vibration experiment expresses that the theoretical result is consistent with the experimental result basically, which indicates that the analysis and data are correct.

## Figures and Tables

**Table 1:** Parameters of positive and negative beams of planar two-dimensional quasi-zero-stiffness vibration isolator.

Parameters of Negative Stiffness Beam				Parameters of Positive Stiffness Beam		
*L*_1_	$w_{1}$	$t_{1}$	*h*	*L*_2_	$w_{2}$	$t_{2}$
40 mm	10 mm	1 mm	10 mm	13.5 mm	10 mm	1 mm

**Table 2:** Parameters of the material.

	Elastic Modulus E/MPa	Poisson’s Ratio μ	$\mathrm{Density}\;\rho /kg\times m^{-3}$
PP	220	0.35	900
PLA	1400	0.2	1250

## Data Availability

The data supporting the conclusions of this article will be made available by the authors on request.
